# The Copper Chaperone Protein Gene *GmATX1* Promotes Seed Vigor and Seedling Tolerance under Heavy Metal and High Temperature and Humidity Stresses in Transgenic *Arabidopsis*

**DOI:** 10.3390/plants11101325

**Published:** 2022-05-17

**Authors:** Yingzi Shen, Jiaping Wei, Shuang Wang, Xi Zhang, Kebing Mu, Sushuang Liu, Hao Ma

**Affiliations:** 1 State Key Laboratory of Crop Genetics and Germplasm Enhancement, Nanjing Agricultural University, Nanjing 210095, China; 2018201050@stu.njau.edu.cn (Y.S.); weijp@gsau.edu.cn (J.W.); ws1229@126.com (S.W.); 2019201058@njau.edu.cn (X.Z.); 2018101090@njau.edu.cn (K.M.); liu@zjhzu.edu.cn (S.L.); 2 Gansu Province Key Laboratory of Aridland Crop Science, Gansu Agricultural University, Lanzhou 730070, China; 3 Department of Life Science and Health, Huzhou University, Huzhou 313000, China

**Keywords:** *GmATX1*, high temperature and high humidity stress, heavy metal, seed development, seed vigor, tolerance

## Abstract

Abiotic stresses such as high temperature, high humidity, and heavy metals are important factors that affect seed development and quality, and restrict yield in soybean. The ATX1-type copper chaperones are an important type of proteins that are used for maintaining intracellular copper ion homeostasis. In our previous study, a copper chaperone protein *GmATX1* was identified in developing seeds of soybean under high temperature and humidity (HTH) stresses. In this study, the *GmATX1* gene was isolated, and multiple alignment analysis showed that its encoding protein shared high sequence identities with other plant orthologues of copper chaperone proteins containing the HMA domain, and a conserved metal ion-binding site, CXXC. A subcellular localization assay indicated that *GmATX1* was localized in the cell membrane and nucleus. An expression analysis indicated that *GmATX1* was involved in seed development, and in response to HTH and heavy metal stresses in soybean. *GmATX1*-silent soybean seedlings were found to be more severely damaged than the control under HTH stress. Moreover, the silencing of *GmATX1* reduced antioxidase activity and reactive oxygen species (ROS) scavenging ability in the seedling leaves. The overexpression of *GmATX1* in *Arabidopsis* improved seed vigor and seedling tolerance, and enhanced antioxidase activity and ROS scavenging ability under HTH and heavy metal stresses. Our results indicated that *GmATX1* could promote seed vigor and seedling tolerance to HTH and heavy metal stresses in transgenic *Arabidopsis*, and this promotion could be achieved by enhancing the antioxidase activity and ROS scavenging ability.

## 1. Introduction

Soybean (*Glycine max* (L.) Merr.) is the world’s major protein and oil seed crop, providing two-thirds of the calories derived from agriculture [1,2]. However, soybean seeds easily deteriorate during development, maturity, and storage, due to their high protein and oil contents [3]. The phenomenon of pre-harvest seed deterioration occurs frequently in many soybean production regions around the world [4,5]. It is considered that high temperature and humidity (HTH) stresses during the physiological maturity period (R7 stage) of soybean seeds in the field is a main factor that leads to pre-harvest seed deterioration [6,7]. This deterioration can result in a decrease in seed vigor and nutritional quality, ultimately constraining yield [7].

As a catalytic or structural cofactor, copper is involved in various physiological and developmental processes during plant growth and development, including photosynthesis, respiratory metabolism, cell wall reconstruction, and biotic and abiotic stress responses [8,9,10,11,12]. Visible symptoms of copper deficiency include stunted growth, distortion or whitening of young leaves, damage to the apical meristem, and decreased seed setting and yield [9,13,14]. Excess copper, however, is extremely toxic to plants and generates reactive oxygen species (ROS), causing cellular damage [6]. For example, when copper ions are excessive, they will trigger a series of redox reactions in cells, producing a large amount of ROS and inducing lipid peroxidation, which will cause damage to DNA, intracellular macromolecules, and the structure and function of cells [9,13]. To deal with the damage of excessive copper, plants have formed a sophisticated regulatory system to maintain the balance of copper ions in cells. In this supervisory system, copper chaperone protein plays a key role.

Copper chaperone proteins are a set of low molecular weight metal transporters that are involved in intracellular copper transport. They transport copper to specific copper receptors and copper-dependent enzymes [15,16]. The copper chaperone protein was first found in brewing yeast. Later, copper chaperone proteins were also noted in plants. It has been found that these plant copper chaperone proteins are homologous to yeast copper chaperone proteins, and they all contain a copper-binding region, which maintains the copper balance in cells by binding copper. In plants, there exist two kind of ATX-like copper chaperone proteins, ATX1-type and CCH-type, which are associated with intracellular copper transport and ROS production. So far, many functions of ATX1-type copper chaperone proteins have been explored. For example, the overexpression of the *AtATX1* gene improves tolerance to copper overload in *Arabidopsis* and increases plant susceptibility to copper deficiency under conditions of severe copper deficiency [17,18]. The *NtATX1* gene is involved in the transport and distribution of both copper and Cd in tobacco. The *ATX* gene in rice is not only induced by hormones such as salicylic acid, abscisic acid, and jasmonic acid, but also in response to pathogenic bacteria [19]. The *OsATX1* gene plays an important role in the regulation of copper transport, facilitating the transport of copper from roots to stems in rice, and is responsible for the redistribution of copper from old leaves to developing tissues and seeds. All of these results suggest that copper chaperone proteins play important roles in the tolerance of plants to adverse environments or pathogens.

In one of our previous studies, a key copper chaperone protein, *GmATX1*, was identified in the seeds using differential proteomic analysis of leaves, cotyledons, and embryos of the pre-harvest seed deterioration-sensitive soybean cultivar Ningzhen No. 1 and -resistant cultivar Xiangdou No. 3 during seed development under HTH stress [20]. In the present study, the *GmATX1* gene was isolated and characterized. qRT-PCR was used to investigate the expression patterns of *GmATX1* in various tissues, and to verify whether *GmATX1* was involved in the response to HTH stress in soybean or not. The *GmATX1*-silent soybean line and the *GmATX1*-overexpressed *Arabidopsis* line were generated and used to investigate a series of physiological, biochemical, and morphological traits, including antioxidase activity, lipid peroxidation, ROS accumulation, seed germination, and viability, etc. The results will enable us to better understand the functions of *GmATX1* proteins in plants, and to provide potential genes for improving seed vigor.

## 2. Results

### 2.1. Sequence Characterization and Subcellular Location of *GmATX1*

**Figure 1:**
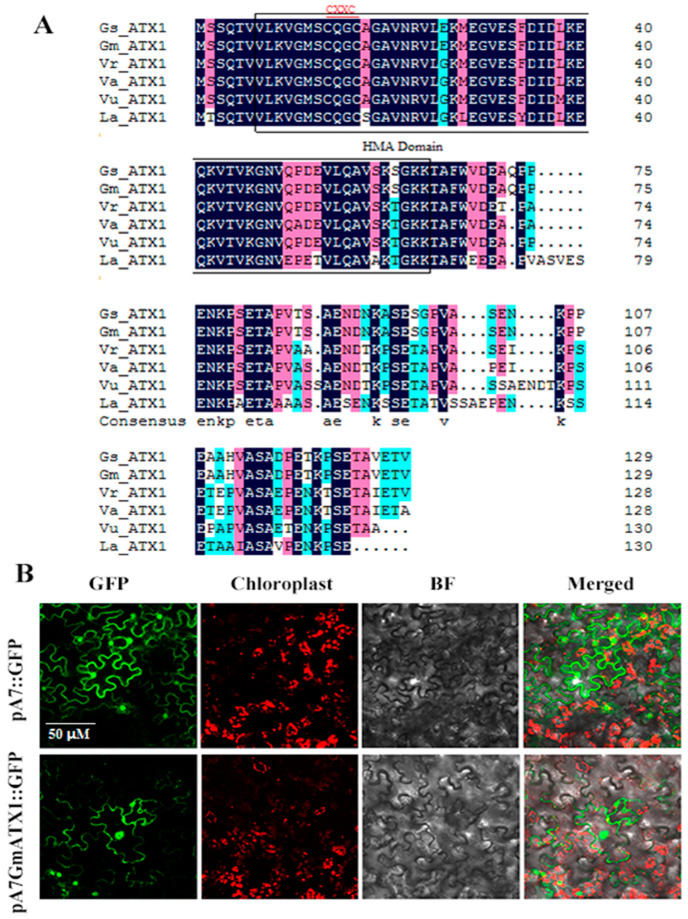
Sequence analysis and subcellular localization of *GmATX1*. (**A**) Multiple alignment of the amino acid sequences of *GmATX1* and other plant homologues. CXXC motif, heavy metal binding core motif. Gs, *Glycine soja*; Gm, *Glycine max*; Vr, *Vigna radiata*; Va, *Vigna angularis*; Vu, *Vigna unguiculata*; La, *Lupinus angustifolius*. (**B**) Subcellular localization of the *GmATX1* protein in tobacco leaf cells. pA7*GmATX1*::GFP, a fusion protein; pA7*GmATX1*::GFP, a null-loaded protein; BF, Brightfield. Fluorescence images of protoplasts expressing GFP fusion proteins were obtained using the LSM780 confocal microscopy imaging system.

The integral cDNA of *GmATX1* (Gene ID: 100305622, NCBI) was obtained from the leaves of soybean cultivar Xiangdou No. 3, and contained an open reading frame of 390 bp, encoding a peptide of 130 amino acids. Multiple amino acid sequence alignment analysis demonstrated that the *GmATX1* protein shared high sequence identities with other orthologues of copper chaperone proteins in plants, and all of them contained the HMA domain and a conserved metal ion binding site CXXC (Figure 1A).

To determine the subcellular localization of *GmATX1*, a plasmid containing the *GmATX1*-GFP fusion protein gene was constructed and transiently expressed in tobacco leaf cells. The *GmATX1*-GFP signal was found to be concentrated in the nucleus and cell membrane (Figure 1B), indicating that *GmATX1* was localized in the nucleus and the cell membrane.

### 2.2. *GmATX1* Expression in Various Tissues and under HTH Stress in Soybean

**Figure 2:**
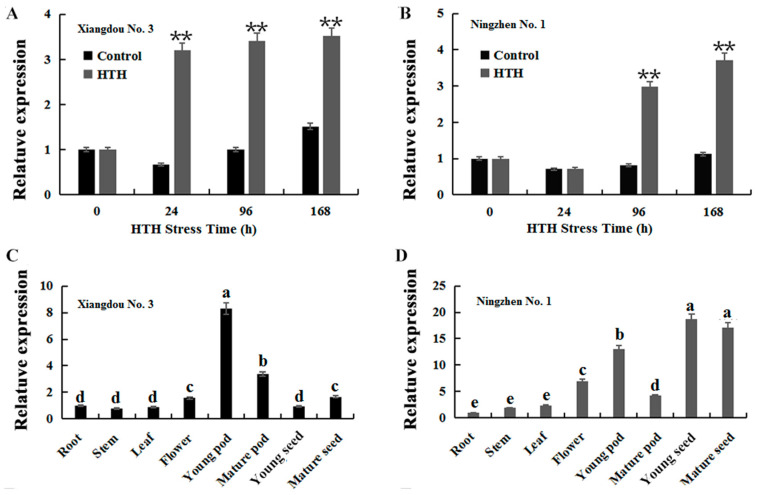
Expression patterns of *GmATX1* in soybean. (**A**,**B**) Expression patterns of *GmATX1* in the developing seeds of soybean cvs. Xiangdou No. 3 and Ningzhen No. 1 at the R7 stage, treated with the HTH stress for 0, 24, 96, and 168 h. (**C**,**D**) Expression of *GmATX1* in various tissues in soybean cvs. Xiangdou No. 3 and Ningzhen No. 1 under normal growth and development conditions. Young seed, developing seed at the R5 stage (beginning of bulge); mature seed, seed at the R7 stage (maturity). ** Indicates significant differences between the control and the treatment at *p* < 0.01; different letters within a cultivar indicate significant differences at *p* < 0.05. Values are mean ± SD from three biological replicates.

The expression levels of *GmATX1* in developing seeds were evaluated using the soybean cultivars (cvs.) Ningzhen No. 1 and Xiangdou No. 3 at the R7 stage under HTH treatment. The results showed that compared to those of the controls, the expression levels of *GmATX1* in the developing seeds were very significantly (*p* < 0.01) increased at all of the stress time points in the resistant cultivar Xiangdou No. 3, and only at the stress time points of 96 and 168 h in the sensitive cultivar Ningzhen No. 1 (Figure 2A,B). These results indicated that *GmATX1* was involved in the response to HTH stress in the developing seeds of soybean.

*GmATX1* was found to display various expression levels in different tissues of soybean. The highest expression level of *GmATX1* was found in pods, followed by in mature seeds and flowers in Xiangdou No. 3 (Figure 2C), while in seeds, followed by in young pods and flowers in Ningzhen No. 1 (Figure 2D). Only a small amount of expression was detected in the stems, leaves, and roots of both the soybean cultivars. These results indicated that *GmATX1* might be mainly involved in seed development in soybean.

### 2.3. *GmATX1* Enhancement of Seedling Tolerance, Antioxidase Activity, and ROS Scavenging Ability in Soybean under HT and HTH Stresses

**Figure 3:**
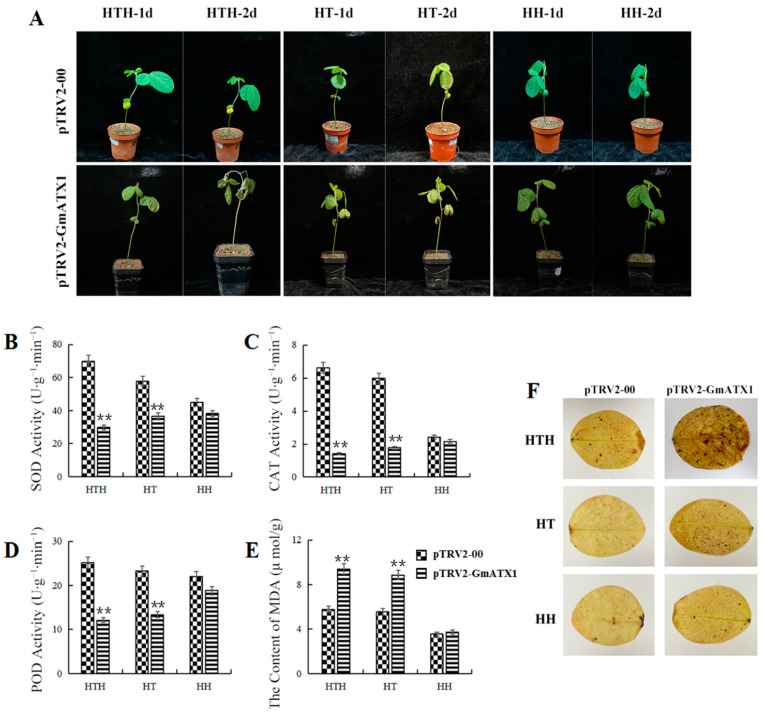
Effects of HT, HH, and HTH stresses on the *GmATX1*-silent soybean line. (**A**) Illustration of the 5-week-old seedlings of the *GmATX1*-silent soybean line (pTRV2-*GmATX1*) and control (pTRV2-00) treated under HT (40/30 °C, 10 h/14 h (light/dark) and 70% relative humidity (RH)), HH (30/20 °C, 10 h/14 h (light/dark) and 100% RH), and HTH (40/30 °C, 10 h/14 h (light/dark) and 100% RH) condition, respectively, for 2 d. (**B**–**E**) Activities of SOD, CAT, and POD and the content of MDA in the leaves of 5-week-old seedlings of the gene-silenced soybean line (pTRV2-*GmATX1*) and control (pTRV2-00) treated under HT, HH, and HTH conditions, respectively, for 2 d. (**F**) Staining of H_2_O_2_ in the leaves of 5-week-old seedlings of the gene-silenced soybean line (pTRV2-*GmATX1*) and the control (pTRV2-00) treated under the HT, HH, and HTH conditions, respectively, for 2 d. The values shown are mean ± SD from three biological replicates. ** Indicates significant differences between the *GmATX1*-silent soybean line (pTRV2-*GmATX1*) and the control (pTRV2-00) at *p* < 0.01.

To assess the effect of *GmATX1* in response to high humidity (HH), high temperature (HT), and HTH stresses, the soybean line with *GmATX1*-silent (pTRV2-*GmATX1*) was generated. Under the HTH stress, the *GmATX1*-silent seedlings showed wilting and yellowing, while the control seedlings only slight yellowing at the edges of leaves. Under HT stress, the gene-silent seedlings showed more yellowing than the control seedlings. However, no significant change in phenotype was found under HH stress between the gene silent seedlings and the control seedlings (Figure 3A). The results indicated that *GmATX1* could enhance the tolerance of soybean seedlings to HT and HTH stresses.

To further clarify the physiological and biochemical basis of *GmATX1* function in promoting stress tolerance, 5-week-old seedlings of the *GmATX1*-silent soybean line (pTRV2-*GmATX1*), treated under HTH, HT, and HH conditions, respectively, were used. The results indicated that compared to those of the control, the activities of catalase (CAT), superoxide dismutase (SOD), and peroxidase (POD) in the seedling leaves of the gene-silenced soybean line were significantly (*p* < 0.01) reduced (Figure 3B–D), whereas the malondialdehyde (MDA) contents were significantly (*p* < 0.01) elevated under the HT and HTH treatments (Figure 3E). Furthermore, higher levels of hydrogen peroxide (H_2_O_2_) were also found in the leaves of the gene-silenced soybean line (Figure 3F). However, the antioxidase activities in the seedling leaves of the *GmATX1*-silent soybean line were decreased, but not to a significant level under the HH condition, and the MDA content and H_2_O_2_ levels were increased, but not to a significant level. These results implied that *GmATX1* could enhance antioxidase activity and ROS scavenging ability, leading to an enhanced tolerance to HT and HTH stresses in soybean.

### 2.4. *GmATX1* Overexpression in Arabidopsis Promoting Seed Vitality, Seedling Tolerance, Antioxidase Activity, and ROS Scavenging Ability under HT and HTH Stresses

**Figure 4:**
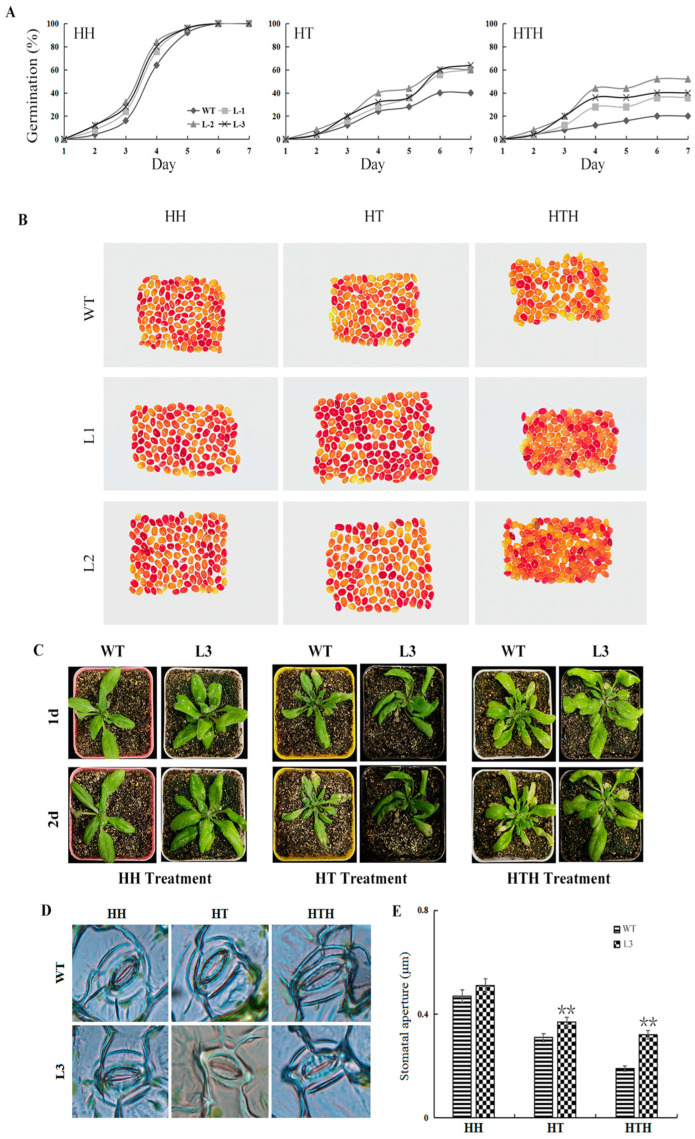
Phenotype, seed germination, and viability of WT and *GmATX1*-overexpressed *Arabidopsis* lines under HT, HH, and HTH treatments. (**A**) Germination percentages of seeds harvested from the WT and *GmATX1*-overexpressed *Arabidopsis* lines (L1, L2 and L3) after the HT (40/20 °C, 12 h/12 h (light/dark) and 70% RH), HH (23/20 °C, 12 h/12 h (light/dark) and 100% RH), and HTH (40/20 °C, 12 h/12 h (light/dark) and 100% RH) treatments, respectively. (**B**) Viability of the seeds harvested from the WT and transgenic *Arabidopsis* lines (L1 and L2) after the HT, HH, and HTH treatments, respectively. Dark red staining indicates viable seeds. (**C**) Illustration of the 4-week-old WT and transgenic *Arabidopsis* line (L3) after the HT, HH, and HTH treatments, respectively, for 2 d. (**D**,**E**) Stomatal morphology and stomatal aperture of the 4-week-old WT and transgenic *Arabidopsis* lines (L3) under the HH, HTH, and HT stresses, respectively. Values are mean ± SD from three biological replicates. ** indicates significant differences between the WT and transgenic *Arabidopsis* lines (L3) at *p* < 0.01.

Germination of the seeds harvested from the WT and the *GmATX1*-overexpressed *Arabidopsis* lines (L1, L2, and L3) was investigated after HH, HT, and HTH treatment, respectively. It was worth noticing that the seeds of the transgenic *Arabidopsis* lines possessed higher (*p* < 0.05) germination percentages than those of the WT after the HT and HTH treatments, except for after the HH treatment (Table 1, Figure 4A). However, after the HH treatment, the transgenic *Arabidopsis* lines possessed faster germination rates than the WT. The 2,3,5-triphenyltetrazolium chloride (TTC) staining assay demonstrated that more (*p* < 0.05) seeds were viable in the transgenic *Arabidopsis* lines (L1 and L2) than in the WT after the HH, HT, and HTH stresses (Table 2, Figure 4B). Interestingly, the seed-staining rates (%) after the HTH stress were lower (*p* < 0.05) than those after the HT and HH stresses in the WT and the transgenic *Arabidopsis* lines (Table 2), indicating that the seeds under HTH stress possessed a more severe impact on seed vitality than those with the HH and HT stresses.

Furthermore, the 4-week-old seedlings of the *GmATX1*-overexpressed *Arabidopsis* line (L3) were assessed for their tolerance to HH, HT, and HTH stresses. After treatment for 2 d under the HTH and HT conditions, respectively, the WT plants were more seriously damaged than the transgenic plants, showing more serious chlorosis or more severe wilting (Figure 4C). Moreover, the stomata of the WT plants were nearly completely closed, while the transgenic plants had greater (*p* < 0.01) stomatal opening than the WT plants under the HTH and HT stresses (Figure 4D,E). However, no significant difference in morphology changes was found between the WT and the transgenic *Arabidopsis* lines under the HH stress. Taken together, all of the above results demonstrated that the overexpression of *GmATX1* in *Arabidopsis* could enhance the germination percentage and the vitality of seeds and the tolerance of seedlings under HT and HTH stresses.

**Figure 5:**
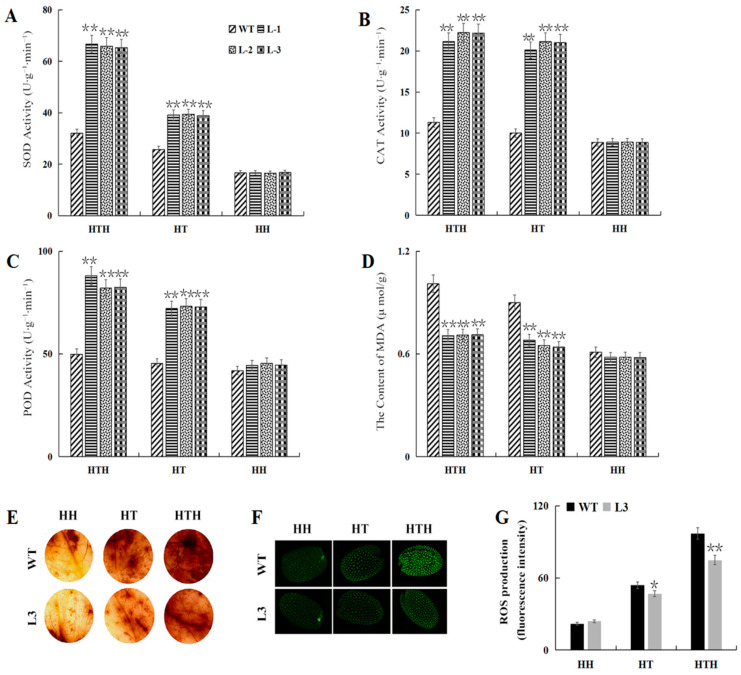
Effects of HT, HH, and HTH stresses on antioxidase activity and ROS scavenging ability of the WT and *GmATX1*-overexpressed *Arabidopsis* lines. (**A**–**D**) Activities of SOD, CAT, and POD and the content of MDA in the leaves of the 4-week-old seedlings of the WT and transgenic *Arabidopsis* lines (L1, L2, and L3) after the HT, HH, and HTH treatments, respectively, for 2 d. (**E**) Staining of H_2_O_2_ in the leaves of the 4-week-old seedlings of the WT and transgenic *Arabidopsis* lines (L3) after the HT, HH, and HTH treatments, respectively, for 2 d. (**F**,**G**) ROS levels in the seeds harvested from the WT and transgenic *Arabidopsis* (L3) after the HT, HH, and HTH treatments, respectively, for 2 d. The values shown are mean ± SD from three biological replicates. * and ** indicate significant differences between the WT and transgenic *Arabidopsis* lines at *p* < 0.05 and *p* < 0.01, respectively.

To understand the physiological and biochemical basis of tolerance, antioxidase activity and lipid peroxidation in the leaves of the 4-week-old seedlings of the *GmATX1*-overexpression *Arabidopsis* lines (L1, L2, and L3) under different (HH, HT, and HTH) stresses were further investigated. Compared to those of the WT, the activities of SOD, CAT, and POD in the leaves of the *GmATX1*-overexpressed *Arabidopsis* lines were significantly (*p* < 0.01) elevated under the HT and HTH stresses (Figure 5A–C); but the MDA contents in the leaves of the transgenic *Arabidopsis* lines were significantly (*p* < 0.01) lower than in those of the WT (Figure 5D). Moreover, higher levels of H_2_O_2_ were also found in the leaves of the WT plants than in those of the transgenic *Arabidopsis* line (L3) (Figure 5E). Similar results were observed in the H2DCFDA staining of seeds under the HT and HTH treatments, which indicated that the accumulation of ROS was seriously (*p* < 0.05 or *p* < 0.01) reduced in the transgenic *Arabidopsis* lines under the HT and HTH stresses (Figure 5F,G). However, no significant (*p* > 0.05) difference was found in antioxidase activity, lipid peroxidation, or ROS accumulation between the WT and the transgenic *Arabidopsis* lines under the HH stress. Taken together, all of the above results implied that the overexpression of *GmATX1* could enhance antioxidase activity and ROS scavenging ability under HTH and HT stresses, thus enhancing the tolerance to them in transgenic *Arabidopsis*.

### 2.5. *GmATX1* Enhances Seedling Tolerance and Antioxidase Activity in Arabidopsis under Heavy Metal Stress

**Figure 6:**
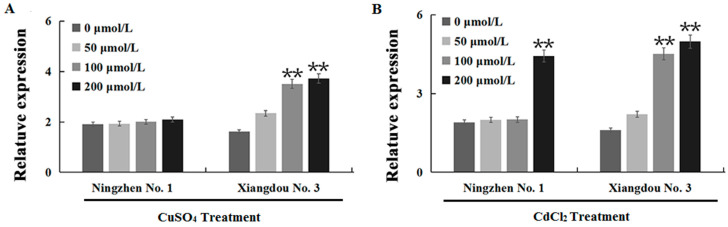
Expression pattern of *GmATX1* in soybean cvs. Ningzhen No. 1 and Xiangdou No. 3 under heavy metal stress. (**A**) The relative expression of *GmATX1* under different concentrations of CuSO_4_ stress (for 24 h) in roots of 2-week-old soybean seedlings. (**B**) The relative expression of *GmATX1* under different concentrations of CdCl_2_ stress (for 24 h) in roots of 2-week-old soybean seedlings. ** Indicates significant differences between the control and treatments at *p* < 0.01. Values are mean ± SD from three biological replicates.

Since *GmATX1* belongs to copper chaperone proteins, it is necessary to understand whether it is involved in the tolerance to heavy metals in plants. In the present study, firstly, the expression of *GmATX1* in soybean seedling roots treated with different concentrations (0, 50, 100, and 200 μmol/L) of CuSO_4_ and CdCl_2_, respectively, was analyzed using qRT-PCR. Compared to the controls, the level of *GmATX1* was significantly (*p* < 0.01) elevated in the roots of the soybean cultivar Xiangdou No. 1, with an increase in the concentration of CuSO_4_ and CdCl_2_, while it significantly (*p* < 0.01) accumulated in the roots of soybean cultivar Ningzhen No. 1 only with an increase in the concentration of CdCl_2_ (Figure 6A,B). These results indicated that *GmATX1* might be involved in the response to the coercion of heavy metals (CuSO_4_ and CdCl_2_) in plants.

**Figure 7:**
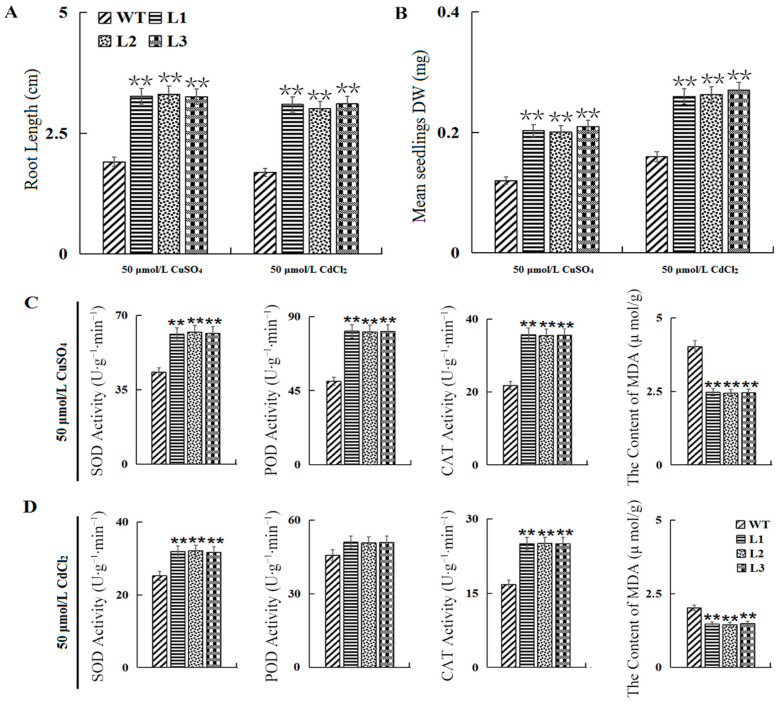
Effects of heavy metal stresses on antioxidase activity and seedling tolerance of WT and *GmATX1*-overexpressed *Arabidopsis*. (**A**) Effect on the root length of the transgenic *Arabidopsis* seedlings after sowing on MS medium containing 50 μmol/L CuSO_4_ and 50 μmol/L CdCl_2_, respectively, for 10 d. (**B**) Effect on DW of the transgenic *Arabidopsis* seedlings after sowing on MS medium containing 50 μmol/L CuSO_4_ and 50 μmol/L CdCl_2_, respectively, for 10 d. (**C**) Activities of SOD, CAT, and POD, and contents of MDA in the leaves of seedlings of the transgenic *Arabidopsis* lines (L1, L2, and L3) and WT after sowing on MS medium containing 50 μmol/L CuSO_4_ for 10 d. (**D**) Activities of SOD, CAT, and POD, and contents of MDA in the leaves of the seedlings of transgenic *Arabidopsis* lines (L1, L2, and L3) and WT after sowing on MS medium containing 50 μmol/L CdCl_2_ for 10 d. Values shown are mean ± SD from three biological replicates. ** Indicates significant differences at *p* < 0.01.

Secondly, the tolerance of the seedlings of the *GmATX1*-overexpressed *Arabidopsis* lines to CuSO_4_ and CdCl_2_ stresses was investigated. After sowing on MS medium containing 50 μmol/L CuSO_4_ and 50 μmol/L CdCl_2_, respectively, for 10 d, the transgenic *Arabidopsis* seedlings were found to show significantly (*p* < 0.01) longer roots than the WT (Figure 7A); and the dry weight (DW) of the transgenic *Arabidopsis* seedlings was also significantly (*p* < 0.01) higher than that of the WT (Figure 7B). The results suggest that the overexpression of *GmATX1* in *Arabidopsis* could enhance the tolerance of seedlings to heavy metal (CuSO_4_ and CdCl_2_) stresses.

Moreover, antioxidase activity and lipid peroxidation in the leaves of the seedlings of the transgenic *Arabidopsis* lines under CuSO_4_ and CdCl_2_ treatment, respectively, were investigated. Compared to those of the WT, the activities of SOD and CAT in the leaves of the *GmATX1*-overexpressed *Arabidopsis* lines (L1, L2, and L3) were significantly (*p* < 0.01) elevated under the CuSO_4_ and CdCl_2_ treatments, while the activities of POD were significantly (*p* < 0.01) raised only under CuSO_4_ stress (Figure 7C,D). The MDA contents in the leaves of the *GmATX1*-overexpressed *Arabidopsis* lines were significantly (*p* < 0.01) lower than in those of the WT under the CuSO_4_ and CdCl_2_ treatments (Figure 7C,D). The results implied that the overexpression of *GmATX1* in *Arabidopsis* could enhance antioxidase activity and ROS scavenging ability under CuSO_4_ and CdCl_2_ stresses, thus enhancing the tolerance of plants to CuSO_4_ and CdCl_2_ stresses.

## 3. Discussion

Metal chaperone proteins are able to transport metal ions to specific organelles for isolation or excretion by binding tightly to them, thus protecting the plant from heavy metal toxicity [21,22]. Although the function of the copper chaperone in dicotyledonous and monocotyledonous plants has been studied to some extent [23], there has been little research on its response to HTH stress in soybean. In our previous proteomics study, a copper chaperone protein named *GmATX1* was identified in the developing seeds of soybean under HTH stress [20]. In the present study, the *GmATX1* gene was isolated from soybean and characterized. The *GmATX1* protein contained a conserved metal ion-binding sequence of CXXC (Figure 1A), which is common to bacteria, animals, and plants, suggesting a distinct evolutionary conservation of ATX1 proteins, and its importance in the copper-trafficking process and the maintenance of copper homeostasis [19,24]. Subcellular localization demonstrated that *GmATX1* specifically localized to the nucleus and cell membrane (Figure 1B). The qRT-PCR results showed that *GmATX1* was expressed highly in developing seeds under normal growth conditions (Figure 2C,D) and under HTH stress (Figure 2A,B). Through gene silencing technology, *GmATX1* was found to be able to enhance the tolerance of soybean seedlings to HTH stresses (Figure 3A). By transgenic technology, the seedlings of *GmATX1*-overexpressed *Arabidopsis* lines showed more tolerance to HTH stress than those of the control (Figure 4C–E). All of the results indicated that *GmATX1* was involved in the response to HTH stress and could enhance plant tolerance to HTH stress. In addition, the overexpression of *GmATX1* in *Arabidopsis* was found to be able to significantly (*p* < 0.05) promote seed germination percentage and vitality under HTH stress (Table 1 and Table 2, Figure 4A,B). These results indicated that *GmATX1* might play an important role in seed vigor formation under HTH stress in plants.

ROS are a class of toxic products that are produced in plants under adverse conditions, mainly O^2−^ and H_2_O_2_. ROS production induced by adverse environmental stimuli is a common phenomenon in plants [25]. On one hand, plants can respond to various stresses through ROS to adapt to the environment, but on the other hand, excessive ROS in plants will cause damage to cell structure and function, enzyme systems, and DNA, etc. [26,27]. In plant cells, ROS can be scavenged through several major ROS-scavenging enzymes, such as SOD, CAT, and POD, and these ROS-scavenging enzymes are crucial for their tolerance to various stresses in plants [28,29]. Peroxidase activity in plant cells is usually detected using 3,3′-diaminobenzidine (DAB) staining, which can visually reflect the damaged state of the plant by staining the leaf shades. In the present study, the antioxidant enzyme activity and ROS scavenging ability in the *GmATX1*-silent soybean seedlings under HTH stress were significantly (*p* < 0.01) reduced, and their tolerance to the stress was reduced (Figure 3). However, in *GmATX1*-overexpressed *Arabidopsis* lines under HTH stress, antioxidant enzyme activity and ROS scavenging ability were significantly (*p* < 0.05 or *p* < 0.01) enhanced in the seedling leaves and the developing seeds, and their seedling tolerance to stress and seed vitality were promoted (Table 1 and Table 2, Figure 4A,B, and Figure 5A–E). All of these results indicated that *GmATX1* could promote seed vigor and seedling tolerance to HTH stress, through enhancing antioxidase activity and ROS scavenging ability in plants. However, the molecular mechanism of how *GmATX1* works needs further study.

In addition, *GmATX1* was found to be able to enhance the tolerance of the seedlings of soybean and transgenic *Arabidopsis* to HT stress (Figure 3A and Figure 4C–E), and to significantly (*p* < 0.05) promote seed vitality under HT stress (Table 1 and Table 2, Figure 4A,B). Moreover, it could significantly (*p* < 0.05 or *p* < 0.01) enhance antioxidase activity and ROS scavenging ability under HT stress in plants (Table 1 and Table 2, Figure 3, Figure 4A,B and Figure 5A–E). However, *GmATX1* could significantly (*p* < 0.05) promote seed vitality in transgenic *Arabidopsis* under HH stress (Table 2), but had no significant (*p* > 0.05) effects on the morphology, physiology, or biochemistry of seedlings (Table 1, Figure 3, Figure 4 and Figure 5). Interestingly, HT stress alone and the HT stress combined with the HH (namely, HTH) stress were found to cause various changes in plants, but the HH stress led to no change, except for seed vitality in transgenic *Arabidopsis*. The results might be due to the fact that HH stress must work by interacting with HT stress, which was supported by data showing that the effect of HTH stress on plants was greater than that of HT stress in this study.

Unprecedented bioaccumulation and biomagnification of heavy metals in the environment have become a dilemma for all living organisms, including plants. For example, cadmium stress has been found to be able to inhibit the seed germination of soybean, mung bean, and other leguminous crops [30]. Additionally, copper is the fourth largest source of heavy metal pollution on farmland in China. Copper in excess can enhance the production of ROS, which cause cell damage and affect plant growth, especially root growth, with significant inhibitory effects, ultimately causing yield loss [31,32]. Copper chaperone proteins in plants have been found to play important roles in maintaining the copper balance in cells by binding copper and enhancing tolerance to adverse environments or pathogens. The expression of *GmATX1*, a copper chaperone protein gene, was also found to be higher (*p* < 0.01) under CdCl_2_ (>50 μmol/L) and CuSO_4_ (> 50 μmol/L) stresses than under the control condition, in soybean cvs. Ningzhen No. 1 and Xiangdou No. 3, except for under CuSO_4_ treatment in the cultivar Ningzhen No. 1 (Figure 6). The results indicated that *GmATX1* was also involved in the response to heavy metal stress in soybean. Moreover, the root length and the dry weight of the *Arabidopsis* seedlings overexpressing *GmATX1* were found to be significantly (*p* < 0.01) higher than those of the WT after treatment with 50 μmol/L CuSO_4_ and 50 μmol/L CdCl_2_, respectively (Figure 7A,B). Additionally, the overexpression of *GmATX1* in *Arabidopsis* significantly (*p* < 0.01) increased SOD, CAT, and POD activities, and markedly (*p* < 0.01) decreased lipid peroxidation in seedling leaves under CuSO_4_ and CdCl_2_ stresses, except for POD activity under CdCl_2_ stress (Figure 7C,D). All of these results imply that *GmATX1* could enhance plant tolerance to copper and cadmium stresses through promoting antioxidase activity and ROS scavenging ability. However, the molecular mechanism of how *GmATX1* deals with heavy metals, and the differences in the molecular mechanism of how *GmATX1* responds to heavy metals and HTH stresses, needs further study. Taken together, our results imply that *GmATX1* might be a potential gene that is used in genetic engineering for the enhancement of seed vigor and stress tolerance.

Gene expression and function are influenced by genetic background. In the present study, compared to those of the controls, the expression levels of *GmATX1* in the developing seeds were found to be very significantly (*p* < 0.01) increased at all of the stress time points in the pre-harvest seed deterioration-resistant cultivar Xiangdou No. 3, and only at the stress time points of 96 and 168 h in the sensitive cultivar Ningzhen No. 1 (Figure 2A,B). The highest expression level of *GmATX1* was found in pods, followed by mature seeds and flowers in Xiangdou No. 3 (Figure 2C), while the highest expression level was found in seeds, followed by young pods and flowers in Ningzhen No. 1 (Figure 2D). However, no difference in the promoter and coding regions of *GmATX1* has been found between the two soybean cultivars (data not shown). Therefore, the expression differences of *GmATX1* under HTH stress and in different tissues between the two soybean cultivars might be due to differences between their genetics backgrounds. In addition, the increased expression of *GmATX1* in soybean seedlings under 50 µmol/L CdCl_2_ and 50 µmol/L CuSO_4_ treatment, respectively, was found to be not significant (*p* > 0.05), but the same concentration of copper and cadmium influenced root growth in transgenic *Arabidopsis* lines. The results might be caused by the efficiency of heavy metal uptake by plants being related to soil characteristics and plant species, which requires further experiments for elucidation.

## 4. Materials and Methods

### 4.1. Isolation of *GmATX1* and Subcellular Localization

The integral cDNA sequence of *GmATX1* was obtained from a cDNA library generated from matured soybean seeds. *GmATX1* was isolated from the leaves of the soybean cultivar Xiangdou No. 3, using a pair of gene-specific primers, *GmATX1*-F and *GmATX1*-R (Supplementary Table S1). For subcellular location, its integral cDNA sequence was amplified via PCR using a pair of gene-specific primers, pA7-*GmATX1*-F and pA7-*GmATX1*-R (Supplementary Table S1), and cloned into an empty pA7 plasmid, which contained a green fluorescent protein (GFP) symbol. The PCR conditions were as follows: 95 °C for 5 min and 30 cycles of 94 °C for 45 s, 56 °C for 45 s, and 72 °C for 120 s, with a final extension at 72 °C for 10 min. The suspension liquid containing the recombinant plasmid was injected into the leaves of 4-week-old tobacco plants, followed by incubation at 28 °C for 1 d in the dark, and 2 d in 16 h of light. The confocal microscopy imaging system (Zeiss LSM780, Jena, Germany) was used to monitor the GFP localization. A 543 nm laser was used to detect the spontaneous fluorescence of the chloroplast in tobacco mesophyll cells, while a 488 nm laser was used to activate the fluorescence of GFP.

### 4.2. Generation of the *GmATX1*-Silent Soybean Line

The specific region of the *GmATX1* gene was searched according to NCBI BLAST and its primers were designed (Supplementary Table S1). The recovered amplified DNA fragment was digested using the restriction endonucleases *Eco*R I and *Bam*H I, and inserted into the pTRV2 vector. The pTRV2-*GmATX1* vector was transferred into *Agrobacterium tumefaciens* strain GV3101. Soybean plants with the first true leaves removed were selected for the experiment and divided into the following two groups: (1) a negative control group, where pTRV2 and pTRV1 empty vectors were mixed at a 1:1 ratio and injected into the plants; and (2) an experimental group, in which pTRV2-*GmATX1* and pTRV1 empty vectors were mixed at a 1:1 ratio into a water suspension with which plants were watered every five days. The plants were then cultivated in an intelligent growth chamber with the following conditions: 20/13 °C (day/night) temperature, 10/14 h (day/night) photoperiod, and 60% RH. The method of obtaining *GmPDS*-silent plants was the same as above. Three weeks later, leaves were collected for RNA extraction. In this study, the tobacco fragility virus (TRV) was used as the vector, and *GmPDS* was used as a positive control [33]. The first round of compound leaves and the second round of compound leaves showed obvious whitening 7 d after the last watering of the infection solution (Supplementary Figure S1A,B), indicating that the pTRV1 and pTRV2 carriers used in this experiment could be used in the soybean VIGS silencing study. To ensure the success of VIGS, the expression level of *GmATX1* in the plants infiltrated with pTRV1/pTRV2-*GmATX1* (plants-pTRV2-*GmATX1*) was further analyzed using qRT-PCR. Compared to the plants infiltrated with *A. tumefaciens* carrying pTRV1/pTRV2 (plants-pTRV2-00), the plants infiltrated with *A. tumefaciens* carrying pTRV1/pTRV2-*GmATX1* (plants-pTRV2-*GmATX1*) had a significantly low (*p* < 0.01) expression level of *GmATX1* (Supplementary Figure S1C,D), suggesting the successful inhibition of *GmATX1*. Therefore, the *GmATX1*-silent soybean line (pTRV2-*GmATX1*) was obtained for subsequent experiments.

### 4.3. Generation of *GmATX1*-Overexpressed Arabidopsis

To construct the plant-transforming vector pBI121-*GmATX1*, the open reading frame of *GmATX1* was cloned into the binary plasmid pBI121-GUS (kanamycin resistance) under the control of the CaMV 35S promoter [34]. The primers used in this study are listed in Supplementary Table S1. The pBI121-*GmATX1* vector was transferred into *Agrobacterium* *tumefaciens* strain GV3101, and followed by *Arabidopsis* transformation according to the floral dip method [35]. Potential transgenic seeds were screened under the pressure of 0.05 mg/mL kanamycin. Three *GmATX1*-overexpressing *Arabidopsis* lines (L1, L2, and L3) were obtained for subsequent experiments.

### 4.4. Stress Experiments

A pair of pre-harvest seed deterioration-sensitive and -resistant soybean cvs. Ningzhen No. 1 and Xiangdou No. 3 was used as experimental materials. Seeds were grown in pots and maintained at 30/20 °C, 70% RH, and 10/14 h (light/dark). Potted plants at the R7 stage were subjected to HTH, HT, and HH stresses according to Wang et al. [7]. For the HTH stress, the potted plants were moved into an artificial climate room under 40/30 °C, 100% RH, and 10/14 h cycle (light/dark) for 7 d. For HT stress, the potted plants were moved into an artificial climate room under 40/30 °C, 70% RH, and 10/14 h cycle (light/dark) for 7 d; and for HH stress, the potted plants were moved into an artificial climate room under 30/20 °C, 100% RH, and 10/14 h cycle (light/dark) for 7 d. In parallel, the potted control plants in the equal growth stage were cultivated under normal conditions (30/20 °C, 70% RH, and 10/14 h (light/dark)) for 7 d. For each stress, seeds were collected from the central part of 10 control plants and 10 treated plants of each soybean cultivar after being treated for 24, 96, and 168 h, respectively, and each sample contained three independent biological replicates. The time points were selected according to our previous study, and at these time points, the developing soybean seeds were found to respond strongly to HTH stress at the morphological, physiological and biochemical, and molecular levels [7]. All of the collected samples were refrigerated in liquid nitrogen and kept subsequently in an ultra-low temperature refrigerator for total RNA extraction.

Soybean seeds were germinated on germination paper and transferred to hydroponic nutrient solution for 14 d. Seedlings of uniform growth were transferred to hydroponic nutrient solution containing 0 (blank control), 50, 100, and 200 μmol/L CuSO_4_ and CdCl_2_, respectively, for 24 h. The root systems of 10 soybean seedlings from each treatment were selected for expression pattern analysis. In addition, the seeds of the WT and the T3 generation of the *GmATX1*-overexpressed *Arabidopsis* lines were sown on MS medium containing CuSO_4_ and CdCl_2_, respectively, germinated, and grown for 10 d. The root length, dry weight (DW), antioxidase activity, lipid peroxidation, and ROS levels of the seedlings were analyzed.

The plants of the WT and the *GmATX1*-overexpressed *Arabidopsis* lines at physiological maturity stage and at the seedling stage were subjected to HTH, HT, and HH treatments, respectively. For HTH stress, the *Arabidopsis* lines were moved into an artificial climate room under 40/20 °C, 100% RH, and a 12/12 h cycle (light/dark) for 2 d. For HT stress, the *Arabidopsis* lines were subjected to 40/20 °C, 70% RH, and a 12/12 h cycle (light/dark) for 2 d. For HH stress, the *Arabidopsis* lines were subjected to 23/20 °C, 100% RH, and a 12/12 h cycle (light/dark) for 2 d.

### 4.5. Total RNA Extraction and qRT-PCR Analysis

Total RNA was extracted from different tissues or organs of soybeans using the Universal Plant RNA Extraction Kit (Vazyme Biotech, Beijing, China). Synthesis of cDNA was performed using the PrimeScript^TM^ RT reagent kit (TaKaRa, Dalian, China). The qRT-PCR was performed using the SYBR Green Real-time PCR Master Mix (TaKaRa, Dalian, China), and the relative gene expression levels were calculated using the comparative C_T_ method [36].

The transcript levels of *GmATX1* in the *GmATX1*-silent soybean line were analyzed using qRT-PCR (Supplementary Figure S1), and the soybean *Actin* gene (Gene ID: 100798052, NCBI) was used as an internal control. All primers used in the study were listed in Supplementary Table S1.

### 4.6. Germination and TTC Assay

The plants of the WT and the overexpressed *Arabidopsis* lines at the physiological maturity stage were cultivated under the control, HT, HH, and HTH conditions, respectively, for 2 d. After the stress, the developing seeds were harvested separately and used for germination assays. The germination assay was conducted in a growth cabinet at 23/20 °C, with a photoperiod of 12 h/12 h (light/dark). From the first to seventh days after sowing, the germination numbers of *Arabidopsis thaliana* were recorded every 24 h. For the TTC assay, harvested seeds were immersed in 1% (*w/v*) 2, 3, 5-triphenyltetrazolium chloride solution at 30 °C in darkness for 2 d. These staining seeds were observed using the MVX10 stereo fluorescence microscope (Olympus, Tokyo, Japan). Dark red staining indicated viable seeds. Each experiment was conducted with three independent biological replicates.

### 4.7. Phenotype and Stomatal Morphology Analysis of *GmATX1*-Overexpressed Arabidopsis

The 4-week-old seedlings of WT and transgenic *Arabidopsis* lines were treated for 2 d under the HT, HH, and HTH conditions, respectively. A MVX10 stereo fluorescence microscope (Olympus, Tokyo, Japan) was used to examine the stomatal characteristics. The stomatal aperture was counted using the Software Image J (version 1.8.0). All of the experiments were repeated at least three times, and the results from one representative experiment were shown.

### 4.8. Antioxidase Activity and Lipid Peroxidation Assay

The leaves collected from the 4-week-old seedlings of the WT, *GmATX1*-overexpressed *Arabidopsis* lines (L1, L2, and L3), and the 5-week-old *GmATX1*-silent soybean line (pTRV2-*GmATX1*) under HT, HH, and HTH treatments for 2 d, respectively, were used in the determination of SOD, CAT, and POD activities, and lipid peroxidation. An antioxidase activity assay was performed according to Song et al. [37], with some modifications. Briefly, the samples were well homogenized in extraction buffer at 4 °C, which consisted of 50 mM pH 7.8 sodium phosphate buffer and 1 mM EDTA-Na_2_, as well as 1% (weight/volume) polyvinyl pyrrolidone. The homogenates were centrifuged at 4 °C for 15 min at 10,000× *g*, and then supernatants were used for the analysis of CAT, SOD, and POD activities using a Thermo Multiscan FC. The level of lipid peroxidation was assessed by measuring malondialdehyde (MDA) content according to Chu et al. [38], with minor modifications. The samples were fully ground and extracted in a buffer of 0.67% TBA in 20% TCA at 4 °C, and then centrifuged at 10,000× *g* for 20 min. The absorbance of each sample supernatant was measured at 450, 532, and 600 nm. All measurements were replicated three times.

### 4.9. Hydrogen Peroxide (H_2_O_2_) Staining and ROS Release Assay

H_2_O_2_ staining was conducted according to Mao et al. [39], using the leaves of the seedlings of the WT and *GmATX1*-overexpressed *Arabidopsis* lines (L3) under HH, HT, and HTH treatments for 2 d, respectively. For soybean, the leaves of the 5-week-old seedlings of the *GmATX1*-silent line (pTRV2-*GmATX1*) and the control (pTRV2-00), treated under the HT, HH, and HTH conditions, respectively, for 2 d, were used. Samples were stained with 0.5 mg/mL 3,3′-diaminobenzidine (DAB, Solarbio) buffer, and then incubated in DAB solution at 25 °C for 10 h and bleached with 95% ethanol.

ROS levels in the harvested seeds of WT and *GmATX1*-overexpressed *Arabidopsis* lines (L3) treated with the HH, HT, and HTH for 2 d, respectively, were measured as previously described by Liu et al. [40]. Non-treated plants were used as the control. Briefly, samples were immersed into an incubation solution (10 mM H_2_DCFDA, 0.2 mM CaCl_2_, 10 mM Hepes-NaOH, pH 5.7) at 25 °C in the dark for 30 min, and then washed 3 times in deionized water. The fluorescence intensity of all samples was observed using a LSM780 confocal microscope at 488 nm excitation and at 525 nm emission. Each sample was measured for at least three biological replications.

### 4.10. Statistics Analysis

Data were obtained from the mean value of all replicates. Variance was analyzed using the Student’s *t*-test, and significant differences between samples were indicated with a confidence interval of 99% or 95%.

## 5. Conclusions

The *GmATX1* gene had an open reading frame of 390 bp, encoding a peptide of 130 amino acids. The *GmATX1* protein contained the HMA domain and a conserved metal ion binding site CXXC, similar to other copper chaperone proteins, and was localized in the cell membrane and nucleus. *GmATX1* showed high expression levels in seeds and pods, and in response to HTH stress in soybean. Silencing of *GmATX1* could decrease the tolerance of soybean seedlings to HTH stress and significantly (*p* < 0.01) reduce antioxidase activity and ROS scavenging abilities under HTH stresses. The overexpression of *GmATX1* in *Arabidopsis* significantly (*p* < 0.05) promoted seed germination percentage and vitality under HTH stress, and enhanced seedling tolerance to HTH stress. Moreover, overexpression significantly (*p* < 0.01) promoted antioxidase activity and ROS scavenging ability under HTH stress. Our results indicated that *GmATX1* might play significant roles in seed vigor formation and in response to HTH stress through the enhancement of antioxidase activity and ROS scavenging ability in soybean. Additionally, *GmATX1* was found to be involved in the response to copper and cadmium stresses, and enhance plant tolerance to copper and cadmium stresses through promoting the antioxidase activity and ROS scavenging ability. However, this molecular mechanism needs further study.

In addition, *GmATX1* was found to promote seed vigor and seedling tolerance to HT stress, through the enhancement of antioxidase activity and ROS scavenging ability. However, under HH stress, it could promote seed vitality but had no effects on the morphology, physiology, or biochemistry of seedlings.

## Figures and Tables

**Table 1:** Germination rate (%) of seeds harvested from the *GmATX1*-overexpression *Arabidopsis* lines (L1, L2, and L3) and WT after 2 d of different treatments.

Treatments	*Arabidopsis* Lines			
	WT	L1	L2	L3
HH	100	100	100	100
HT	40 b	60 a	60 a	64 a
HTH	20 c	36 b	52 a	40 b

Note: HH, high humidity (23/20 °C, 12 h/12 h (light/dark) and 100% RH); HT, high temperature (40/20 °C, 12 h/12 h (light/dark) and 70% RH); HTH, high temperature and humidity (40/20 °C, 12 h/12 h (light/dark) and 100% RH). Different lowercase letters in the same line indicate significant differences at *p* < 0.05.

**Table 2:** Viability of seeds (%) harvested from the *GmATX1*-overexpression *Arabidopsis* lines (L1 and L2) and WT after 2 d of different treatments.

Treatments	*Arabidopsis* Lines		
	WT	L1	L2
HH	76.38 b	93.64 a	94.55 a
HT	74.74 b	87.23 a	89.36 a
HTH	36.84 b	50.53 a	44.21 a

Note: HH, high humidity (23/20 °C, 12 h/12 h (light/dark) and 100% RH); HT, high temperature (40/20 °C, 12 h/12 h (light/dark) and 70% RH); HTH, high temperature and humidity (40/20 °C, 12 h/12 h (light/dark) and 100% RH). Different lowercase letters in the same line indicate significant differences at *p* < 0.05.

## Data Availability

Not Applicable.

## Supplementary Materials

The following supporting information can be downloaded at: https://www.mdpi.com/article/10.3390/plants11101325/s1, Supplementary Table S1: Sequences of primers used in this study. Supplementary Figure S1: Identification of *GmPDS*-silent and *GmATX1*-silent soybean plants. A, phenotypes of the soybean cultivar Xiangdou No. 3 at 25 d after injection with recombinant viruses carrying *GmPDS*. B, expression of *GmPDS* in the leaves of *GmPDS*-silent soybean. C, phenotypes of the soybean cultivar Xiangdou No. 3, 25 d after injection with recombinant viruses carrying *GmATX1*. D, expression of *GmATX1* in the leaves of the *GmATX1*-silent soybean. The values shown are the mean ± SD from three biological replicates. ** indicates significant differences between the control and the gene-silent soybean line at *p* < 0.01. Supplementary Figure S2: Effect of heavy metal stresses on seedlings of *GmATX1*-overexpressed Arabidopsis. A, effect on root length of the transgenic Arabidopsis seedlings after sown on MS medium containing 50 mol/L CuSO_4_ and 50 mol/L CdCl_2_ for 10 d, respectively. B, effect on dry weight (DW) of the transgenic Arabidopsis seedlings after sown on MS medium containing 50 mol/L CuSO_4_ and 50 mol/L CdCl_2_ for 10 d, respectively. Values shown are mean ± SD from three biological replicates. ** indicates significant differences between the WT and transgenic Ara-bidopsis lines at *p* < 0.01. Supplementary Figure S3: Effects of copper and cadmium stresses on antioxidase activity and ROS scavenging of WT and *GmATX1*-overexpressed Arabidopsis seed-lings. A, activities of SOD, CAT and POD and contents of MDA in the leaves of the seedlings of the transgenic Arabidopsis lines (L1, L2 and L3) and WT after sown on MS medium containing 50 mol/L CuSO_4_ for 10 d; B, activities of SOD, CAT and POD and contents of MDA in the leaves of the seedlings of the transgenic Arabidopsis lines (L1, L2 and L3) and WT after sown on MS me-dium containing 50 mol/L CdCl_2_ for 10 d. Values shown are mean ± SD from three biological replicates. ** indicates significant differences between the WT and transgenic Arabidopsis lines at *p* < 0.01. Supplementary Figure S4: Identification of *GmPDS*-silent and *GmATX1*-silent soy-bean plants. A, phenotypes of soybean cultivar Xiangdou No. 3 at 25 d after injected with re-combinant viruses carrying *GmPDS*. B, expressions of *GmPDS* in leaves of *GmPDS*-silent soy-bean. C, phenotypes of soybean cultivar Xiangdou No. 3 at 25 d after injected with recombinant viruses carrying *GmATX1*. D, expressions of *GmATX1* in leaves of *GmATX1*-silent soybean. Values shown are mean ± SD from three biological replicates. ** indicates significant differences between the control and gene silent soybean line at *p* < 0.01.

## Abbreviations

DAB	Staining, 3,3′-diaminobenzidine staining
ATX1	Copper chaperone protein
BF	Brightfield
Cvs	Cultivars
GFP	Green fluorescent protein
HTH	High temperature and humidity
HT	High temperature
HH	High humidity
ORF	Open reading frame
PCR	Polymerase chain reaction
CAT	Catalase
SOD	Superoxide
POD	Peroxidase
MDA	Malondialdehyde
H_2_O_2_	Hydrogen peroxide
PM	Plasma membrane
qRT-PCR	Quantitative real-time PCR
TTC	2,3,5-Triphenyltetrazolium chloride
RH	Relative humidity
ROS	Reactive oxygen species
WT	Wild type
