# Supplementary material for: The Copper Chaperone Protein Gene GmATX1 Promotes Seed Vigor and Seedling Tolerance under Heavy Metal and High Temperature and Humidity Stresses in Transgenic Arabidopsis
Source: Plants (Basel). 2022 May 17;11(10):1325. doi: 10.3390/plants11101325 (PMC9143580; doi:10.3390/plants11101325)
Supplement: Supplementary file 1 [file plants-11-01325-s001.zip › Supplemental TableS1.pdf]

Supplemental Table S1. Sequences of primers used in this study

Name	Sequence (5'-3')	Function
GmATX1-F	TAAAGACACCACCCTTACACAC	Gene isolation
GmATX1-R	GAAACAGACCTACTTCCACATAGA	Gene isolation
pA7-GmATX1-F	CATTTACGAACGATACTCGAGATGTCTTCTCAGACCGTTG	Subcellular localization assay
pA7-GmATX1-R	CTCGAGTATCGTTCGTAAATGAGCAACAGTTTCAACAGCA	Subcellular localization assay
pBI121-ATX1-F	GAGAACACGGGGGACTCTAGAATGTCTTCTCAGACCGTTG	Transgenic
pBI121-ATX1-R	TCTAGAGTCCCCCGTGTTCTCAGCAACAGTTTCAACAGCA	Transgenic
pBI121-GUS-35s-F	TGAAGATGCCTCTGCCGA	Transgenic
pBI121-GUS-GUS-R	CTGATCGTTAAAACTGCCTGG	Transgenic
TRV2-ATX1-F	GTGAGTAAGGTTACCGAATTCCGGAAAGAAGACGGCATTC	Primers used for VIGS vector construction
TRV2-ATX1-R	CGTGAGCTCGGTACCGGATCCTTTCAACAGCAGTTTCTGA	Primers used for VIGS vector construction
TRV2-GmPDS-F	GTGAGTAAGGTTACCGAATTCTCCACCTTTCGTGCTTCTC	Primers used for VIGS vector construction
TRV2-GmPDS-R	CGTGAGCTCGGTACCGGATCCTCAGGTACGCCCTGCTTTT	Primers used for VIGS vector construction
qRT-GmPDS-F	CGTTTCATTGGACGGGGCTT	Detection of *PDS* relative expression
qRT-GmPDS-R	CAAACGACACGCAAGGGAGA	Detection of *PDS* relative expression
qRT-GmATX1-F	TTCTCAGACCGTTGTCCTCA	Detection of *ATX1* relative expression
qRT-GmATX1-R	CACATAGAAAAATAGAAC	Detection of *ATX1* relative expression
qRT-GmATX1-F	TTCTCAGACCGTTGTCCTCA	Quantitative real-time PCR expression analysis in soybean
qRT-GmATX1-R	TTCACTGTCACCTTCTGCTCCT	Quantitative real-time PCR expression analysis in soybean
Actin-F	CCTCAACCCAAAGGTCAACAG	Quantitative real-time PCR expression analysis
Actin-R	GACCAGCGAGATCCAAACGAA	Quantitative real-time PCR expression analysis
Tubulin-F	CTCAAGAGGTTCTCAGCAGTA	For genetically functional verification
Tubulin-R	TCACCTTCTTCATCCGCAGTT	For genetically functional verification
