# Supplementary material for: The Copper Chaperone Protein Gene GmATX1 Promotes Seed Vigor and Seedling Tolerance under Heavy Metal and High Temperature and Humidity Stresses in Transgenic Arabidopsis
Source: Plants (Basel). 2022 May 17;11(10):1325. doi: 10.3390/plants11101325 (PMC9143580; doi:10.3390/plants11101325)
Supplement: Supplementary file 1 [file plants-11-01325-s001.zip › Supplementary Figure.pdf]

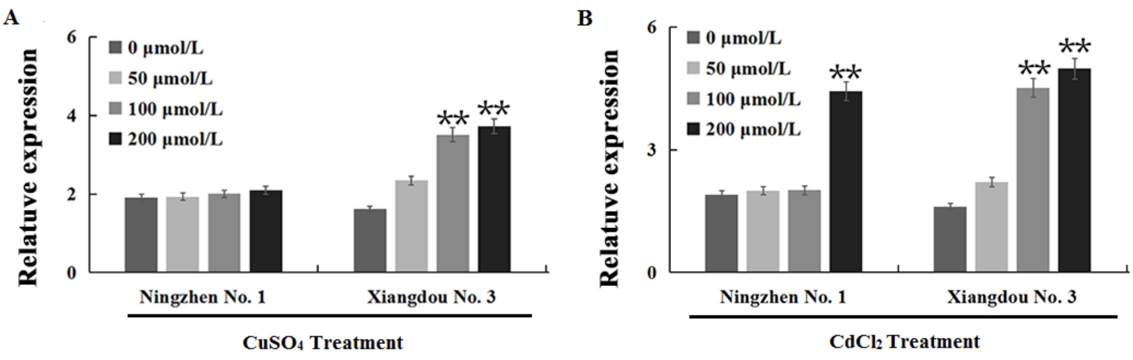

Supplementary Figure S1: Expression pattern of *GmATX1* in soybean cultivars Ningzhen No. 1 and Xiangdou No. 3 under heavy metal stress. **A**, the relative expression of *GmATX1* under different concentration of $CuSO_4$ stress (for 24h) in roots of 2-week-old soybean seedlings. **B**, the relative expression of *GmATX1* under different concentration of $CdCl_2$ stress (for 24h) in roots of 2-week-old soybean seedlings. ** indicates significant differences between the control and treatments at $p < 0.01$. Values are mean ± SD from three biological replicates.

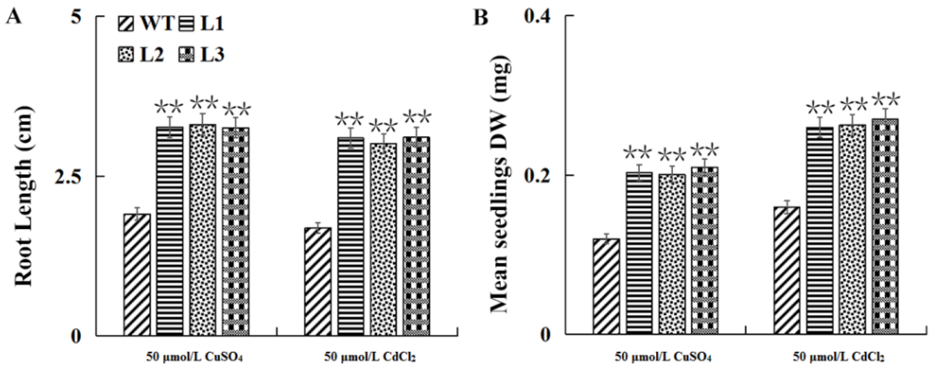

Supplementary Figure S2: Effect of heavy metal stresses on seedlings of *GmATX1*-overexpressed *Arabidopsis.* A, effect on root length of the transgenic *Arabidopsis* seedlings after sown on MS medium containing 50 µmol/L $CuSO_4$ and 50 µmol/L $CdCl_2$ for 10 d, respectively. B, effect on dry weight (DW) of the transgenic *Arabidopsis* seedlings after sown on MS medium containing 50 µmol/L CuSO4 and 50 µmol/L $CdCl_2$ for 10 d, respectively. Values shown are mean ± SD from three biological replicates. ** indicates significant differences between the WT and transgenic *Arabidopsis* lines at $p < 0.01$.

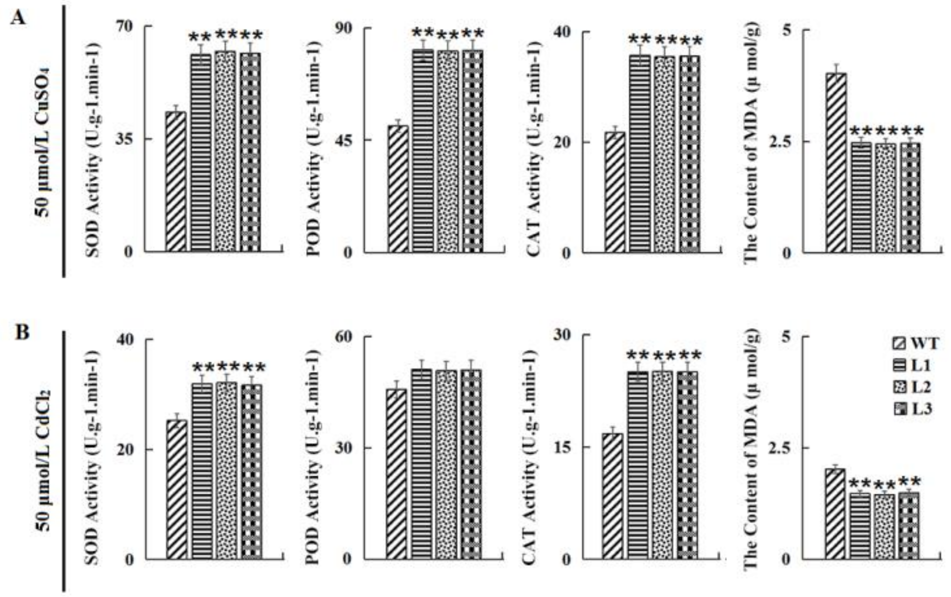

Supplementary Figure S3: Effects of copper and cadmium stresses on antioxidase activity and ROS scavenging of WT and *GmATX1*-overexpressed *Arabidopsis* seedlings. **A**, activities of SOD, CAT and POD and contents of MDA in the leaves of the seedlings of the transgenic *Arabidopsis* lines (L1, L2 and L3) and WT after sown on MS medium containing 50 μmol/L $CuSO_4$ for 10 d; **B**, activities of SOD, CAT and POD and contents of MDA in the leaves of the seedlings of the transgenic *Arabidopsis* lines (L1, L2 and L3) and WT after sown on MS medium containing 50 μmol/L $CdCl_2$ for 10 d. Values shown are mean ± SD from three biological replicates. ** indicates significant differences between the WT and transgenic *Arabidopsis* lines at $p < 0.01$.

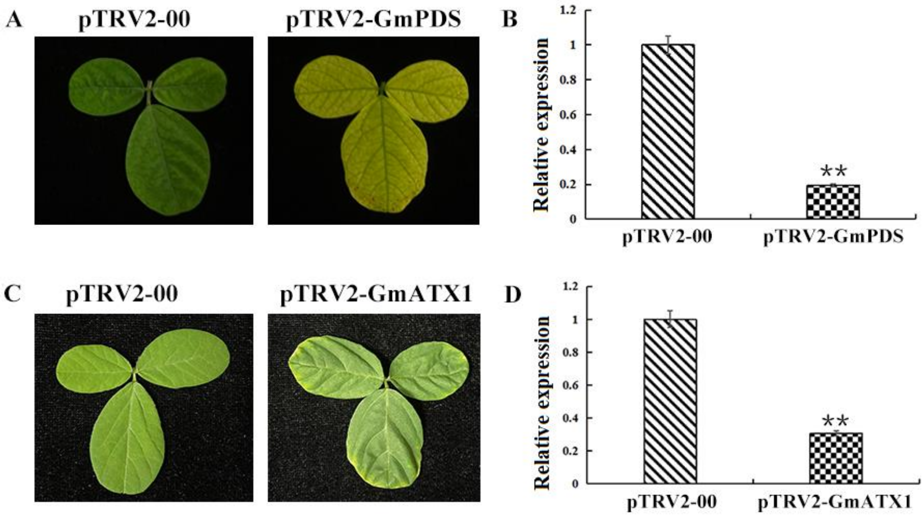

Supplementary Figure S4: Identification of *GmPDS*-silent and *GmATX1*-silent soybean plants. **A**, phenotypes of soybean cultivar Xiangdou No. 3 at 25 d after injected with recombinant viruses carrying

GmPDS. **B**, expressions of *GmPDS* in leaves of *GmPDS*-silent soybean. **C**, phenotypes of soybean cultivar Xiangdou No. 3 at 25 d after injected with recombinant viruses carrying *GmATX1*. **D**, expressions of *GmATX1* in leaves of *GmATX1*-silent soybean**.** Values shown are mean ± SD from three biological replicates. ** indicates significant differences between the control and gene silent soybean line at $p < 0.01$.
